# Mitochondria Clumping vs. Mitochondria Fusion in CMT2A Diseases

**DOI:** 10.3390/life12122110

**Published:** 2022-12-15

**Authors:** Antonietta Franco, Caroline E. Walton, Xiawei Dang

**Affiliations:** 1 Department of Internal Medicine, Washington University School of Medicine, St. Louis, MO 63110, USA; 2 University of North Carolina School of Medicine, Chapel Hill, NC 27599, USA

**Keywords:** mitochondria fitness, mitochondria fusion defects, mitofusin 2, Charcot-Marie-Tooth disease type 2A

## Abstract

Phenotypic variations in Charcot-Marie-Tooth disease type 2A (CMT2A) result from the many mutations in the mitochondrial fusion protein, mitofusin 2 (MFN2). While the GTPase domain mutations of MFN2 lack the ability to hydrolyze GTP and complete mitochondrial fusion, the mechanism of dysfunction in HR1 domain mutations has yet to be explored. Using *Mfn1*/*Mfn2* double null cells and *Mfn2* knock out (KO) fibroblasts, we measured the ability of this variant protein to change conformations and hydrolyze GTP. We found that a mutation in the HR1 domain (M376A) of *MFN2* results in conformational change dysfunction while maintaining GTPase ability. Prolonged exposure to mitofusin agonist MiM 111 reverses mitochondrial fusion dysfunction in the HR1 mutant through encouraging an open conformation, resulting in a potential therapeutic model in this variant. Herein, we describe a novel mechanism of dysfunction in *MFN2* variants through exploring domain-specific mitochondrial characteristics leading to CMT2A.

## 1. Introduction

Mitochondria are double-membraned organelles that regulate cellular integrity, biological process and embryonic development. Notably, they possess highly dynamic morphology regulated by mitochondrial fusion and fission [1]. Mitochondrial fusion occurs at a high rate to exchange mitochondrial content, preserving mitochondrial structure and maintaining membrane potential [1,2,3,4].

In mammalian cells, mitochondrial fusion is mediated by mitofusin 1 (MFN1), mitofusin 2 (MFN2), and optic protein atrophy-1 (OPA1). While OPA1 is an inner membrane protein, mitofusins are GTPase ubiquitinated proteins on the outer mitochondrial membrane [5]. Regulating the first necessary step for mitochondrial fusion, mitofusins form mitochondria-mitochondria tethers mediated by the protein’s conformational changes. Following this, mitochondrial GTPase activity hydrolyzes GTP to GDP, resulting in mitochondrial fusion [6]. 

MFN1 and MFN2 share 80% of their sequences, with the N-terminus and C-terminus domains external to the cytosol, the transmembrane domain inside of the mitochondrial membranes and two coiled-coil heptad-repeat regions (HR1 and HR2) linked by hydrophobic bonds. Besides mitochondrial fusion, MFN2 is involved in tethering mitochondria to the endoplasmic reticulum and releasing calcium [7,8]. MFN2 also recruits Parkin to eliminate damaged mitochondria after being phosphorylated by mitochondrial PTEN-induced putative kinase 1 (PINK1) [3].

Charcot-Marie-Tooth disease type 2A (CMT2A), an untreatable neurodegenerative condition, can result from varying dominant hereditary mutations in *MFN2* [9]. Moreover, human long peripheral and central axons are most sensitive to *MFN2* dysfunction, accounting for the progressive distal weakness and sensory loss associated with CMT2A [10]. We’ve previously reversed mitochondrial fusion dysfunction in a mouse model carrying a human neurodegenerative mitofusin 2 mutation using the fusogenic activity of mitofusin agonists [9].

Approximately 100 different dominant missense mutations in *MFN2* are related to CMT2A, with varying mild, moderate or severe phenotypes. These mutations take place in several mitofusin 2 domains (GTPase, HR1 and HR2), suggesting a necessary investigation into the effect of the location of the domain mutation on the properties of mitochondrial fusion. Loss of function mutations in the *MFN2* GTPase domain, like The105Met and Arg94Glu, have an impaired ability to hydrolyze GTP to GDP, the last step in mitochondrial fusion. This results in shortened and depolarized “clumped mitochondria” that tether together but cannot fuse due to the absence of GTPase activity [11]. It is currently unclear if loss of function mutations in the HR1 domain similarly induces similar perinuclear mitochondrial clumping aggregates with defective GTP catalytic activity. Another possibility is this domain mutation encourages an inactive mitofusin conformation, unable to complete fusion. Here, we investigated and compared the mitochondrial fusion properties between *MFN2* M376A (Met 376 to Ala) in the HR1 domain and *MFN2* T105M (Thr 105 Met) and R94Q (Arg 94 Gln), GTPase mutations. 

The conformation for a closed/inactive mitofusin involves antiparallel intramolecular binding between the coiled-coil heptad-repeat regions HR1 and HR2 [6]. The open/active conformation promotes mitochondrial tethering, which encourages fusion through hydrolysis of GTP to GDP. Based on the crystal structure of bacterial DLP to model the structure of MFN2 [12], mitofusin molecular conformations can be manipulated by peptides to promote an open formation that encourages mitochondrial tethering and fusion [6].

Promoting mitochondrial elongation and fusion, mitofusin agonists can modulate the interactions between HR1 and HR2 domains [13,14,15]. Mitofusin agonists rescue the mitochondrial fusion and motility defects for the *MFN2* T105M mutation, the first therapeutic approach to cure CMT2A in the mouse model. In CMT2A disease, only one copy of *MFN2* is mutated. Mitofusin agonists interact with the two endogenous allele copies for *MFN1* and the one functional copy of *MFN2* to tether and fuse mitochondria together [9]. 

Rocha AG et al. [15] demonstrated that mitofusin 2 allosteric activation is mediated by the specific amino acids Met^376^ and His^380^ in the HR1 domain and Asp^725^ and Leu^727^ in the HR2 domain. We focused on Met^376^ because *MFN2* M376V corresponds to a moderate phenyotype of human CMT2A. *MFN2* M376 to Alanine in the HR1 domain induces a mitochondrial fusion defect, just like *MFN2* R94Q and *MFN2* T105M in the GTPase domain [10,16].

GTPase mutations favor the protein’s closed/inactive conformation, resulting in short mitochondria that lack tethering and fusion abilities [17]. Similarly, mutations in the HR1 domain result in shortened mitochondria that favor the closed/inactive conformation but maintain its GTPase activity. *MFN2* M376A results in a dysfunctional conformation unable to complete mitochondrial fusion. 

Here, we demonstrated:

(1) The source of dysfunction for the *MFN2* M376A mutation in the HR1 domain stems from mitofusin in the inactive conformation, notably maintaining its GTPase abilities.
(2) *MFN2* M376A lacks perinuclear mitochondrial clumping.
(3) Mitofusin agonists MiM 111 and Chimera C open the *MFN2* M376A mutant protein and reverse its dysfunctional mitochondrial fusion abilities.

## 2. Material and Methods

### 2.1. Cell Lines 

*Mfn1*/*Mfn2* double null MEFs and *Mfn2* KO cell fibroblasts were purchased from American Type Culture Collection (ATCC Manassas, VA, USA CRL-2994 and CRL-2993). MEFs were cultured at 37 °C, 5% CO_2_—95% air in Dulbecco’s minimal essential medium (DMEM) containing glucose (4.5 g/L) (Thermo Fisher Scientific, Waltham, MA, USA, 11965-084) with 10% (*v*/*v*) fetal bovine serum (FBS; Gibco, Gaithersburg, MD, USA Cat: # 26140-079), 1× nonessential amino acids (Gibco Cat: # 11130051), 2 mM L-glutamine (Corning, NY, USA. Cat: # 34717007), and 1% (*v*/*v*) penicillin/streptomycin (Gibco Cat: # 15140-122). 

### 2.2. Viral Vectors

*MFN2* mutants and FRET probes were generated using PCR mutagenesis, sequence verified, and sent to Vector BioLabs. Ad-CMV-β-Gal was purchased from Vector BioLab (Cat: #1080).

### 2.3. Protein Structure Modeling

Hypothetical structures of human MFN2 were generated using I-TASSER and Chimera UCSF. The putative closed and open conformation were downloaded (PDB: 2J69 and AF-095140-F1). Domain coloring is as follows: Green GTPase (AA 94-265); Red HR1 (AA 338-421).

### 2.4. Imaging

Live cell studies assessing mitochondrial morphology were performed as described [6,18]. Briefly, MEFs were stained with MitoTracker GREEN (200 nM; Invitrogen, Thermo Fisher Scientific Cat: # M7514) and TMRE (tetramethylrhodamide ethyl ester) ThermoFisher (Cat: # T669). To visualize mitochondrial elongation and depolarization, images were acquired at room temperature on a Nikon Ti Confocal microscope using either a 60 × 1.3 NA oil-immersion objective or 10 × 0.3 NA dry objective, in Krebs–Henseleit buffer (138 nM NaCl, 3.7 nM KCl, 1.2 nM KH_2_PO_4_, 15 nM Glucose, 20 nM HEPES pH: 7.2–7.5, and 1 mM CaCl_2_). Laser excitation was at 488 nm with emission at 510 nm for MitoTracker GREEN and laser excitation was 549 nm with emission at 590 nm for TMRE. Mitochondrial clumping and depolarization were measured as the percentage of cells with clumped mitochondria or depolarized mitochondria, respectively. 

### 2.5. GTPase Activity Assay 

*Mfn1*/*Mfn2* double-null MEFs were transduced with wild type ad-*MFN2*, ad-*MFN2* R94Q, ad-*MFN2* T105M or ad-*MFN2* M376A at multiplicity of infection (M.O.I.) 50. Mitochondria were isolated as described [19], prepared on ice, and used fresh. A total of 100 μg of mitochondrial protein in triplicate was incubated in GTPase Buffer (Promega #V7681; Madison, WI, USA) with 10 μM GTP and 1 mM DTT. Next, 1 μM MiM 111, 1 μM Chimera or DMSO (vehicle) were added as indicated and the reactants were incubated at room temperature for 90 min in 96 well-plates. The Promega GTPase-Glo assay kit was used to measure GTPase activity following the manufacturer’s instructions. Luminescence was quantified on a Promega GloMax Luminometer. 

### 2.6. MFN2 FRET Assays

*Mfn1*/*Mfn2* double-null MEFs were transduced with FRET ad-MFNs. Seventy-two hours later, mitochondria were isolated, and FRET was measured in a 96 well format as described previously [13]. 

### 2.7. Data Presentation and Statistical Analyses

Data are reported as means ± SEM. Sample number (n) indicates the number of independent biological samples. Two-group comparisons used Student’s unpaired *t*-test; multiple group comparisons used one-way ANOVA with Tukey’s post hoc test for individual statistical comparisons. 

## 3. Results

### 3.1. Novel Mechanism of Dysfunction in CMT2A HR1 vs. GTPase Domain Mutation

Most of the severe CMT2A disorders involve mutations in the mitofusin 2 GTPase domain (T105M and R94Q), compromising GTP hydrolysis and impairing mitochondrial fusion. MFN2 and MFN1 are dynamin family GTPases; catalytic GTPase activity is required for mitochondrial fusion, controlling the last/final step in fusing mitochondrial membranes. 

**Figure 1:**
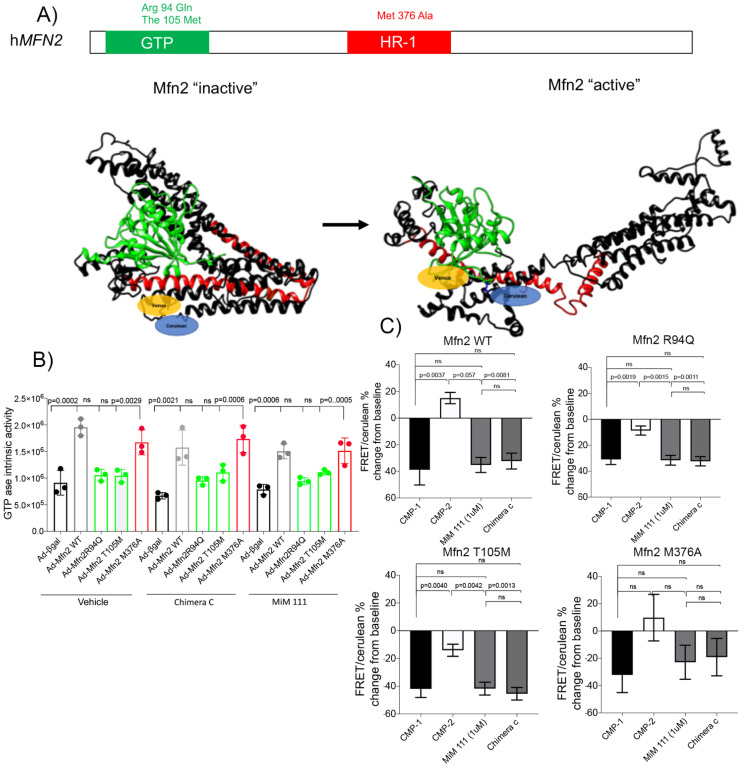
M376A is a conformational mutant but maintains its GTPase ability. (**A**) Human Mitofusin 2 modeling, closed conformation (**left**) and open conformation (**right**). Green GTPase domain (94 to 265 Aa) and Red HR1 domain (338-421 Aa), Venus and Cerulean are the fluorochromes at NT and CT, respectively. (**B**) GTPase activity in mitofusin-null cells on isolated mitochondria, transduced with different adeno-virus mitofusin variant and treated with Vehicle, Chimera C or MiM 111 (1 μM) for 1 h. *p* < 0.05 used ANOVA. (**C**) FRET assay on isolated mitochondria after 4 h of treatment with mitofusin agonist peptides (CMP-1-), mitofusin antagonist peptides (CMP-2) 5 μM, respectively, MiM 111 or Chimera C. (1 μM) *p* < 0.05 used ANOVA for triplicate experiment.

Mutations in the HR1 domain, such as *MFN2* Met^376^Val, mostly results in a moderate phenotype for CMT2A (Figure 1A). In comparing mutations for CMT2A in the GTPase domain (T105M and R94Q) and the HR1 domain (M376A), *MFN2* M376A in the HR1 domain does not impair GTPase activity in mitofusin null cells (Figure 1B).

Based on our previous results [13,14], we demonstrated that mitofusin agonists (Chimera C and MiM 111, also known as Cpd **1** and **2**) break the intramolecular bond in the molecular core of mitofusins, promoting an active conformation capable of tethering. Using FRET mitofusin assay, GTPase defective CMT2A mutants R94Q and T105M retained the ability to change conformations under basal conditions; however, HR1 domain mutant *MFN2* M376A failed to open and remained inactive (Figure 1C). Taken together, the above results reveal a unique constellation of *MFN2* M376A signature: it is the first CMT2A mutation recognized with an impaired ability to change mitofusin conformations while maintaining its GTPase ability.

### 3.2. MFN2 M376A Induces Short Mitochondria without Depolarization

**Figure 2:**
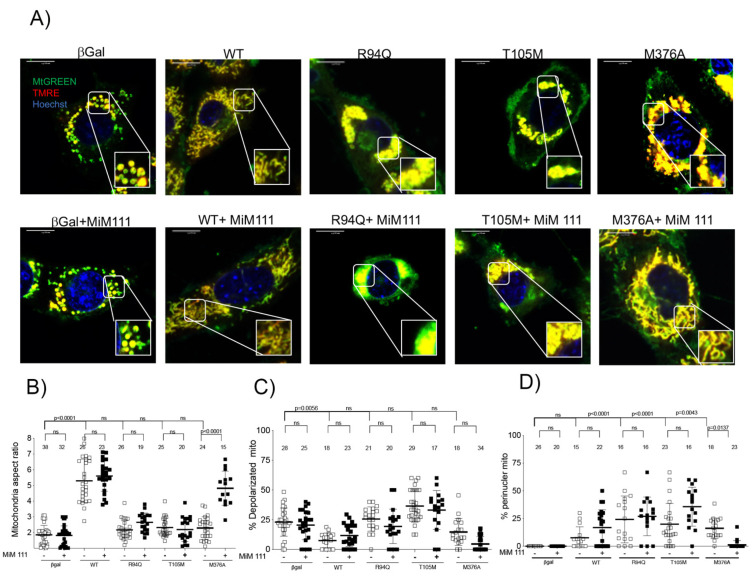
A mitofusin agonist restores mitochondrial fusion dysfunction in HR1 domain mutant in mitofusins null mouse fibroblasts. (**A**) Confocal images in mitofusins null cells stained with MTgreen, TMRE and Hoechst for different adeno-virus for mitofusin mutants in basal condition and after MiM 111 (1 μM). (**B**) Mitochondria aspect ratio with each dot corresponds to single cell for *n* = 3 or 4. Data are means ± SEM, *p* value < 0.05 used 2way-ANOVA. (**C**) Percent depolarized mitochondria with each dot corresponding to single cell for *n* = 3 or 4. Data are means ± SEM, *p* value < 0.05 2way ANOVA. (**D**) Percent perinuclear mitochondria with each dot corresponds to single cell for *n* = 3 or 4. Data are means ± SEM, *p* value < 0.05 2way ANOVA.

To establish the properties of mutant mitochondria, recombinant human adeno Mfn2 expression with different mitofusin variants (T105M, R94Q and M376A) in mitofusins null mouse embryonic fibroblasts were compared to MFN2 WT. All three variants T105M, R94Q and M376A had a reduction in mitochondrial length/width (aspect ratio) (Figure 2A,B), showing that these mutations result in the inability for mitochondria to fuse together compared to the WT.

GTPase mutants R94Q and T105M contain depolarized and aggregated “clumped mitochondria” (Figure 2A,C,D). The HR1 domain mutant M376A does not impact mitochondrial depolarization and lacks perinuclear mitochondrial aggregations (Figure 2A,C,D). The addition of 1 μM of mitofusin activator MiM 111 overnight restored mitochondrial elongation in MFN2 M376A, but did not affect the fusion of the GTPase mutants. Interestingly, MiM 111 reduced perinuclear clumped mitochondria in GTPase mutants in the absence of endogenous mitofusins (Figure 2D). 

### 3.3. Dominant MFN2 M376A Mutation Suppresses Mitochondrial Fusion 

**Figure 3:**
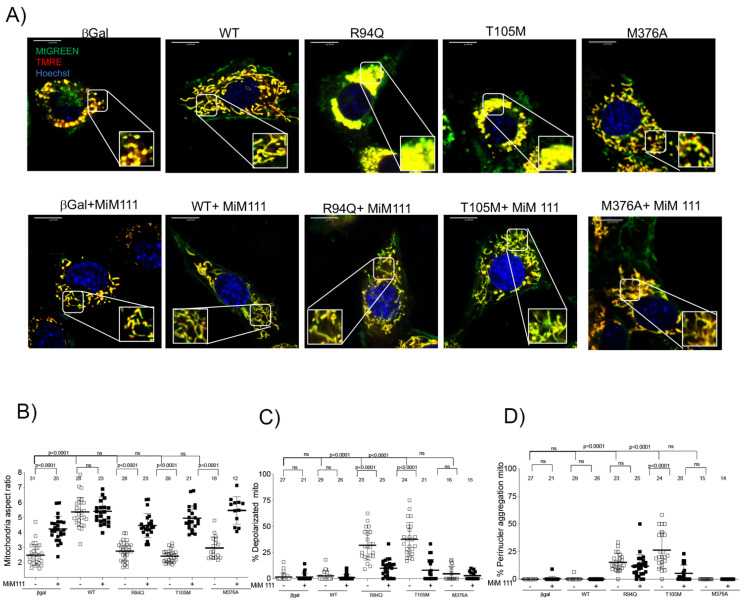
A mitofusin agonist restores mitochondrial fusion dysfunction in HR1 domain mutant in MFN2 null mouse fibroblasts. (**A**) Confocal images in mitofusin 2 KO cells stained with MTgreen, TMRE and Hoechst for different adeno-viri for mitofusin mutants in basal condition and after MiM 111 (1 μM). (**B**) Mitochondrial aspect ratio with each dot corresponding to single cell for *n* = 3 or 4 experiment. Data are mean ± SEM, *p* value < 0.05 2way-ANOVA. (**C**) Percent depolarized mitochondria with each dot corresponding to single cell for *n* = 3 or 4. Data are mean ± SEM, *p* value < 0.05 2way-ANOVA) Percent perinuclear mitochondria with each dot corresponding to single cell for *n* = 3 or 4 experiment. Data are Mean ± SEM, *p* value < 0.05 2way-ANOVA. (**D**) Percent perinuclear mitochondria with each dot corresponds to single cell for *n* = 3 or 4. Data are means ± SEM, *p* value < 0.05 2way ANOVA.

Mitofusin 2 KO cells in the presence of functional endogenous mitofusin 1 retain the dominant mitochondrial fusion defects of the HR1 and GTPase domain mutants. Short mitochondria were observed in T105M, R94Q and M376A (Figure 3B), validating dominant fusion inhibition. Unlike the GTPase mutations, M376A again shows a lack of depolarized mitochondria and perinuclear aggregations (Figure 3A,C,D). 

We can modulate conformational changes in MFN2 using the mitofusin agonist MiM 111 (1 μM overnight). MiM 111 with endogenous mitofusin 1 in mitofusin 2 KO cells, can recover mitochondrial fusion defects and depolarization in cells with the recombinant expression variants (R94Q, T105M and M376A) compared to *MFN2* WT. Furthermore, MiM 111 reduced perinuclear mitochondria clump formation in *MFN2* R94Q and *MFN2* T105M in mitofusin 2 null cells with endogenous mitofusin 1.

Taken together, the above results in both mitofusin null (Figure 2) and *MFN2* KO (Figure 3) reveal the inability of HR1 domain mutants to tether mitochondria under basal conditions. Mitofusin agonists open the mitofusin conformation after prolonged exposure, allowing the mutation to regain its fusion abilities. 

Furthermore, GTPase variants can tether mitochondria together (first step), but are unable to hydrolyze GTP to fuse mitochondria (second step). In T015M and R94Q, mitochondrial clumping was prevalent, consistent with the domain mutation interrupting GTP-dependent mitochondrial fusion, while not interfering with GTPase-independent mitochondrial tethering. Applying 1 µM of mitofusin activator MiM 111 for 24 h in the presence of functional GTPase protein (endogenous mitofusin 1) largely reversed these abnormalities. 

### 3.4. M376A Is a New Shape Shifting Mitofusin 2 Conformational Mutant

**Figure 4:**
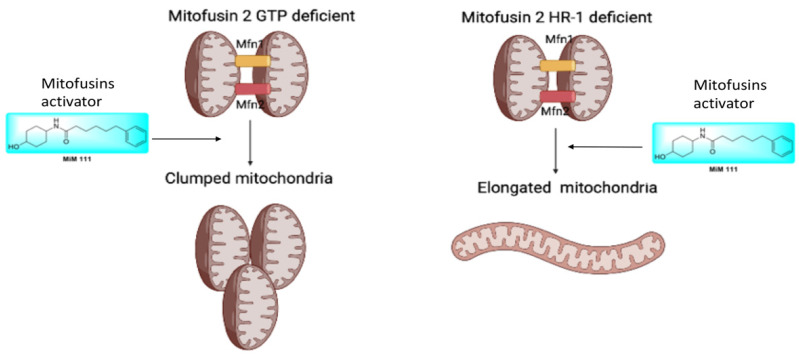
Schematic representation of current mitofusin conformational mechanism. (**Left**) GTPase mitofusin 2 mutants in presence of mitofusin activator (MIM 111) are unable to fuse 2 mitochondria together, forming clumped mitochondrial aggregates. (**Right**) HR1 mitofusin 2 mutants in the presence of mitofusin activators (MIM111) are able to complete mitochondrial fusion.

In the absence of endogenous mitofusin, mitofusin activators are not able to fix mitochondrial fusion defects in *MFN2* R94Q and T105M (GTPase mutants), inducing mitochondrial depolarization and perinuclear aggregates. Based on our evidence of perinuclear mitochondrial clumps, GTPase mutants are able to tether together, but fail to complete mitochondrial fusion in the absence of GTPase activity (Figure 4). In the HR1 domain mutant *MFN2* M376A, the intact GTPase domain can complete mitochondrial fusion in the presence of mitofusin agonists. Thus, mitochondrial fusion is recovered in this mutation. 

## 4. Discussion

Charcot-Marie-Tooth disease type 2A is associated with around 100 different dominant mitofusin 2 mutations, with varying degrees of severity. It has been demonstrated that GTPase domain mutants *MFN2* T105M and R94Q correlate to a severe CMT2A phenotype, while the HR1 mutation *MFN2* M376V results in a moderate phenotype [20]. We compared the mitochondrial properties of mutations in different domains to understand the mechanism of dysfunction associated with mitochondrial fusion. 

Here, we investigated a new *MFN2* mutation at Met^376^, generated using a recombinant adenovirus that resulted in *MFN2* M376A. We focused on M376 because in Rocha AG et al. [15], we demonstrated that mitofusin 2 allosteric activation is mediated by this specific amino acid.

*MFN2* M376A is a shape shifting mitochondrial mutation, favoring its inactive conformation while maintaining its GTPase abilities. This HR1 domain mutation results in short mitochondria, but lacks the perinuclear mitochondrial aggregates associated with GTPase domain mutations. This further affirms that this mutation cannot tether under basal conditions; therefore, it cannot complete mitochondrial fusion. 

Treatment with two structurally distinct but functionally similar allosteric mitofusin activators (Chimera C and MiM 111) yielded no conformational change in the *MFN2* M376A mutant after 4 h but showed that the GTPase activity level was still maintained. We validate that Met^376^ is essential for mitofusin allosteric activation and mutations in the HR1 domain are associated with conformational mitofusin dysfunction instead of GTPase inactivity.

After introducing mitofusin agonist MiM 111 overnight, *MFN2* M376A recovered its mitochondrial fusion abilities, allowing for allosteric modulation. Mitofusin agonists encourage an open conformation in mitofusin null cells expressing recombinant mutants, allowing the functional GTPase to complete mitochondrial fusion in this HR1 domain mutation. This demonstrates that GTPase activity is not compromised by dysfunctional mitofusin conformations. These delayed mitofusin conformational changes with mitofusin agonists represent the first direct evidence to reverse an HR1 domain mutation resulting in CMT2A.

It has been demonstrated previously and here that *MFN2* T105M, R94Q and M376A result in short mitochondria unable to participate in fusion. *MFN2* T105M and R94Q mutations result in depolarized clumped perinuclear mitochondria, able to tether but unable to complete fusion with a lack of GTPase activity. Treatment with the mitofusin activator in a mouse model carrying human *MFN2* T105M can reverse the neuromuscular diseased phenotype [9] acting on both copies of MFN1 and a functional endogenous mitofusin 2 allele.

In the absence of mitofusins, GTPase domain mutants with perinuclear mitochondrial clumps cannot regain its normal mitochondrial fusion abilities through treatment with a mitofusin activator MiM 111. Rather, the number of perinuclear mitochondrial aggregates was reduced and fusion was seen in *MFN2* KO cell with recombinant mitofusin 1 expression after MiM 111 treatment. This suggests that mitofusin 1 GTPase activity was sufficient to fuse mitochondria. 

A limitation of this study is that we substituted alanine onto Met^376^ instead of the human CMT2A mutation which substitutes valine. The presence of valine can compromise the hydrophobic mitofusin core. Because Met^376^ is crucial for mitofusin allosteric activation, studying the domain effects on mitochondrial function at this position was imperative.

More experiments are required to understand the relationship between mitochondrial fusion and tethering. Moreover, these results need to be validated in neuronal cells carrying human CMT2A mutants. Confirming these results may lead to promising mitofusin activator treatment in HR1 domain mutations in CMT2A disease.

## References

[1] Detmer S.A., Chan D.C. (2007). Complementation between mouse Mfn1 and Mfn2 protects mitochondrial fusion defects caused by CMT2A disease mutations. J. Cell Biol..

[2] Friedman J.R., Nunnari J. (2014). Mitochondrial form and function. Nature.

[3] Chen Y., Dorn G.W. (2013). PINK1-phosphorylated mitofusin 2 is a Parkin receptor for culling damaged mitochondria. Science.

[4] Li J., Dang X., Franco A., Dorn G.W. (2022). Reciprocal Regulation of Mitofusin 2-Mediated Mitophagy and Mitochondrial Fusion by Different PINK1 Phosphorylation Events. Front. Cell Dev. Biol..

[5] Dorn G.W. (2019). Evolving Concepts of Mitochondrial Dynamics. Annu. Rev. Physiol..

[6] Franco A., Kitsis R.N., Fleischer J.A., Gavathiotis E., Kornfeld O.S., Gong G., Biris N., Benz A., Qvit N., Donnelly S.K. (2016). Correcting mitochondrial fusion by manipulating mitofusin conformations. Nature.

[7] Kasahara A., Cipolat S., Chen Y., Dorn G.W., Scorrano L. (2013). Mitochondrial fusion directs cardiomyocyte differentiation via calcineurin and Notch signaling. Science.

[8] Ballard A., Zeng R., Zarei A., Shao C., Cox L., Yan H., Franco A., Dorn G.W., Faccio R., Veis D.J. (2020). The tethering function of mitofusin2 controls osteoclast differentiation by modulating the Ca^2+^-NFATc1 axis. J. Biol. Chem..

[9] Franco A., Dang X., Walton E.K., Ho J.N., Zablocka B., Ly C., Miller T.M., Baloh R.H., Shy M.E., Yoo A.S. (2020). Burst mitofusin activation reverses neuromuscular dysfunction in murine CMT2A. Elife.

[10] Larrea D., Pera M., Gonnelli A., Quintana-Cabrera R., Akman H.O., Guardia-Laguarta C., Velasco K.R., Area-Gomez E., Dal Bello F., De Stefani D. (2019). MFN2 mutations in Charcot-Marie-Tooth disease alter mitochondria-associated ER membrane function but do not impair bioenergetics. Hum. Mol. Genet..

[11] Zhou Y., Carmona S., Muhammad A., Bell S., Landeros J., Vazquez M., Ho R., Franco A., Lu B., Dorn G.W. (2019). Restoring mitofusin balance prevents axonal degeneration in a Charcot-Marie-Tooth type 2A model. J. Clin. Investig..

[12] Low H.H., Löwe J. (2006). A bacterial dynamin-like protein. Nature.

[13] Dang X., Zhang L., Franco A., Li J., Rocha A.G., Devanathan S., Dolle R.E., Bernstein P.R., Dorn G.W. (2020). Discovery of 6-Phenylhexanamide Derivatives as Potent Stereoselective Mitofusin Activators for the Treatment of Mitochondrial Diseases. J. Med. Chem..

[14] Dang X., Williams S.B., Devanathan S., Franco A., Fu L., Bernstein P.R., Walters D., Dorn G.W. (2021). Pharmacophore-Based Design of Phenyl-[hydroxycyclohexyl] Cycloalkyl-Carboxamide Mitofusin Activators with Improved Neuronal Activity. J. Med. Chem..

[15] Rocha A.G., Franco A., Krezel A.M., Rumsey J.M., Alberti J.M., Knight W.C., Biris N., Zacharioudakis E., Janetka J.W., Baloh R.H. (2018). MFN2 agonists reverse mitochondrial defects in preclinical models of Charcot-Marie-Tooth disease type 2A. Science.

[16] Dang X., Walton E.K., Zablocka B., Baloh R.H., Shy M.E., Dorn G.W. (2022). Mitochondrial Phenotypes in Genetically Diverse Neurodegenerative Diseases and Their Response to Mitofusin Activation. Cells.

[17] Franco A., Dang X., Zhang L., Molinoff P.B., Dorn G.W. (2022). Mitochondrial Dysfunction and Pharmacodynamics of Mitofusin Activation in Murine Charcot-Marie-Tooth Disease Type 2A. J. Pharmacol. Exp. Ther..

[18] Misko A., Jiang S., Wegorzewska I., Milbrandt J., Baloh R.H. (2010). Mitofusin 2 is necessary for transport of axonal mitochondria and interacts with the Miro/Milton complex. J. Neurosci..

[19] Frezza C., Cipolat S., Scorrano L. (2007). Organelle isolation: Functional mitochondria from mouse liver, muscle and cultured fibroblasts. Nat. Protoc..

[20] Xie Y., Li X., Liu L., Hu Z., Huang S., Zhan Y., Zi X., Xia K., Tang B., Zhang R. (2016). MFN2-related genetic and clinical features in a cohort of Chinese CMT2 patients. J. Peripher. Nerv. Syst..

